# An Acidic Microenvironment Increases NK Cell Killing of *Cryptococcus neoformans* and *Cryptococcus gattii* by Enhancing Perforin Degranulation

**DOI:** 10.1371/journal.ppat.1003439

**Published:** 2013-07-11

**Authors:** Anowara Islam, Shu Shun Li, Paul Oykhman, Martina Timm-McCann, Shaunna M. Huston, Danuta Stack, Richard F. Xiang, Margaret M. Kelly, Christopher H. Mody

**Affiliations:** 1 Department of Microbiology, Immunology and Infectious Diseases, University of Calgary, Calgary, Alberta, Canada; 2 Department of Medical Sciences, University of Calgary, Calgary, Alberta, Canada; 3 Department of Pathology and Laboratory Medicine, University of Calgary, Calgary, Alberta, Canada; 4 Department of Internal Medicine, University of Calgary, Calgary, Alberta, Canada; University of Wisconsin-Madison, United States of America

## Abstract

*Cryptococcus gattii* and *Cryptococcus neoformans* are encapsulated yeasts that can produce a solid tumor-like mass or cryptococcoma. Analogous to malignant tumors, the microenvironment deep within a cryptococcoma is acidic, which presents unique challenges to host defense. Analogous to malignant cells, NK cells kill *Cryptococcus*. Thus, as in tumor defense, NK cells must kill yeast cells across a gradient from physiologic pH to less than 6 in the center of the cryptococcoma. As acidic pH inhibits anti-tumor activities of NK cells, we sought to determine if there was a similar reduction in the anticryptococcal activity of NK cells. Surprisingly, we found that both primary human NK cells and the human NK cell line, YT, have preserved or even enhanced killing of *Cryptococcus* in acidic, compared to physiological, pH. Studies to explore the mechanism of enhanced killing revealed that acidic pH does not increase the effector to target ratio, binding of cytolytic cells to *Cryptococcus*, or the active perforin content in effector cells. By contrast, perforin degranulation was greater at acidic pH, and increased degranulation was preceded by enhanced ERK1/2 phosphorylation, which is essential for killing. Moreover, using a replication defective *ras1* knockout strain of *Cryptococcus* increased degranulation occurred during more rapid replication of the organisms. Finally, NK cells were found intimately associated with *C. gattii* within the cryptococcoma of a fatal infection. These results suggest that NK cells have amplified signaling, degranulation, and greater killing at low pH and when the organisms are replicating quickly, which would help maintain microbicidal host defense despite an acidic microenvironment.

## Author Summary

Immune responses that protect from infection must occur in a variety of unique and potentially hostile environments. Within these environments, acidosis causes profound affects on protective responses. Low pH can occur in focal tumor-like infections, such as in a cryptococcoma produced by the fungal pathogen *Cryptococcus*. Similarly, low pH occurs in focal malignant tumors. It follows that *Cryptococcus* and malignant cells can both be killed by NK cells, which provide an important mechanism of host defense. Thus, we asked whether low pH, which impairs tumor killing, might also affect NK cell killing of *Cryptococcus*. Surprisingly, despite impaired tumor killing, NK cells possess enhanced killing of *Cryptococcus* at low pH. The mechanism involved a gain in intracellular signal transduction that led to enhanced perforin degranulation. This led us to examine NK cells in persistent cryptococcoma of a fatal brain infection and lung. We found that NK cells associate with *Cryptococcus* within the cryptococcoma, but perforin is reduced. These studies suggest NK cell cytotoxicity need not be impaired at low pH, and that enhanced signal transduction and degranulation at low pH might be used to enhance host defense.

## Introduction

The yeast, *Cryptococcus* causes potentially life threatening pneumonia and meningitis. While *C. neoformans* causes infections more commonly in immunosuppressed individuals such as those with AIDS or hematologic malignancies [1], the tropical fungus *C. gattii* has recently emerged on Vancouver Island and the pacific northwest of the United States, where it causes respiratory and meningeal disease in otherwise healthy individuals resulting in disability and even death [2]. Both species produce solid tumor-like lesions called cryptococcomas, although they are somewhat more common in *C. gattii* disease [3], [4]. Cryptococcomas are large focal collections of organisms with infiltrating macrophages and lymphocytes, among other cells [5]. One study reported the presence of lung and brain cryptococcoma in 48% and 18% of cryptococcosis patients, respectively [3]. Unfortunately, the management of cryptococcoma is difficult as they respond poorly to antifungal therapy and sometimes requires surgery to remove the mass due to a space occupying effect in the brain or other tissue [3]. It is not understood why these patients fail to clear these lesions despite possessing a competent immune system; however, the speculation is that unique environmental factors within the cryptococcoma impair the immune response against this fungus. These observations have led us to explore the influence of microenvironmental factors on immune recognition and killing of this pathogen.

Cryptococcal host defense is complex and many cells, including NK cells, contribute to optimal clearance [6]–[8]. NK cells are large granular lymphocytes that directly kill tumor cells, allografts, virally infected cells and microbes [9]–[12]. Studies have established the importance of NK cells in host defense against *Cryptococcus* [6]–[8]. Since NK cells kill tumor cells as well as *Cryptococcus*, we wondered whether the same challenges faced by NK cells in the microenvironment of a tumor might also limit the activity of NK cells in the microenvironment of a cryptococcoma.

*In vivo* studies performed in animal models showed that the pH within the center of a brain cryptococcoma is as low as pH 5.6 [13]. The acidification of the cryptococcoma is believed to result from production of acetate by the organisms, which lowers the pH [14]. Thus, there is a gradient from physiological pH (pH = 7.34–7.4) at the periphery to a pH as low as 5.6 in the center of the cryptococcoma [13]. Similarly, the pH of human and animal tumors ranges between pH 5.6 to 7.2 as a result of glycolysis stimulated by hypoxia, which occurs due to inefficient perfusion resulting from malformed vasculature [15], [16]. Consequently, immune cells may be challenged to recognize and kill both malignant cells and microbes across a gradient from physiologic pH to a pH as low as 5.6.

Prior studies revealed that acidic extracellular pH inhibits the cytotoxicity of human NK cells against a variety of tumor cells [17], [18]. Acidic pH impairs NK cell killing of K562 erythroleukemia cells, which is predominantly mediated via granule exocytosis and release of perforin and granzymes [17]. In other studies, the influence of an acidic microenvironment on the antitumor activity of mouse NK cells using YAC-1 lymphoma cells reported a similar inhibitory effect of acidic pH [19]. Lysis of these tumor cells was significantly reduced at pH 6.4 and 6.7 compared to pH 7.4. Acidic pH was also shown to decrease the cytotoxicity of a murine T lymphocyte clone against syngeneic and allogeneic target cells [20]. Therefore, the acidic pH-mediated inhibition of lymphocyte cytotoxicity of tumor cells is considered to be one of the mechanisms by which tumor cells evade the immune system. Thus, we wondered if NK cells were similarly affected in the acidic microenvironment of a cryptococcoma and responsible for impaired host defense.

To address these questions, the anticryptococcal activity of the human NK cell line, YT, or primary NK cells was tested at physiologic and acidic pH. The ability of YT cells to kill *Cryptococcus* was confirmed by a limiting dilution-based microwell assay as well as permeability to propidium iodide. To explore the mechanism of enhanced anticryptococcal activity in acidic pH, binding of the organism was assessed by detecting conjugates using a flow cytometric assay and downstream signaling was assessed by Erk1/2 phosphorylation, which has previously been shown to be essential for cryptococcal killing by NK cells [21]. In separate assays, the influence of pH and active replication using a replication defective *ras1* mutant on the perforin content and degranulation was assessed by flow cytometric techniques. Finally, histopathologic sections were examined to determine the presence and cytotoxic potential of NK cells in a cryptococcoma.

## Results

### NK cell-mediated anti-cryptococcal activity is enhanced in acidic pH, while anti-tumor activity is decreased

**Figure 1:**
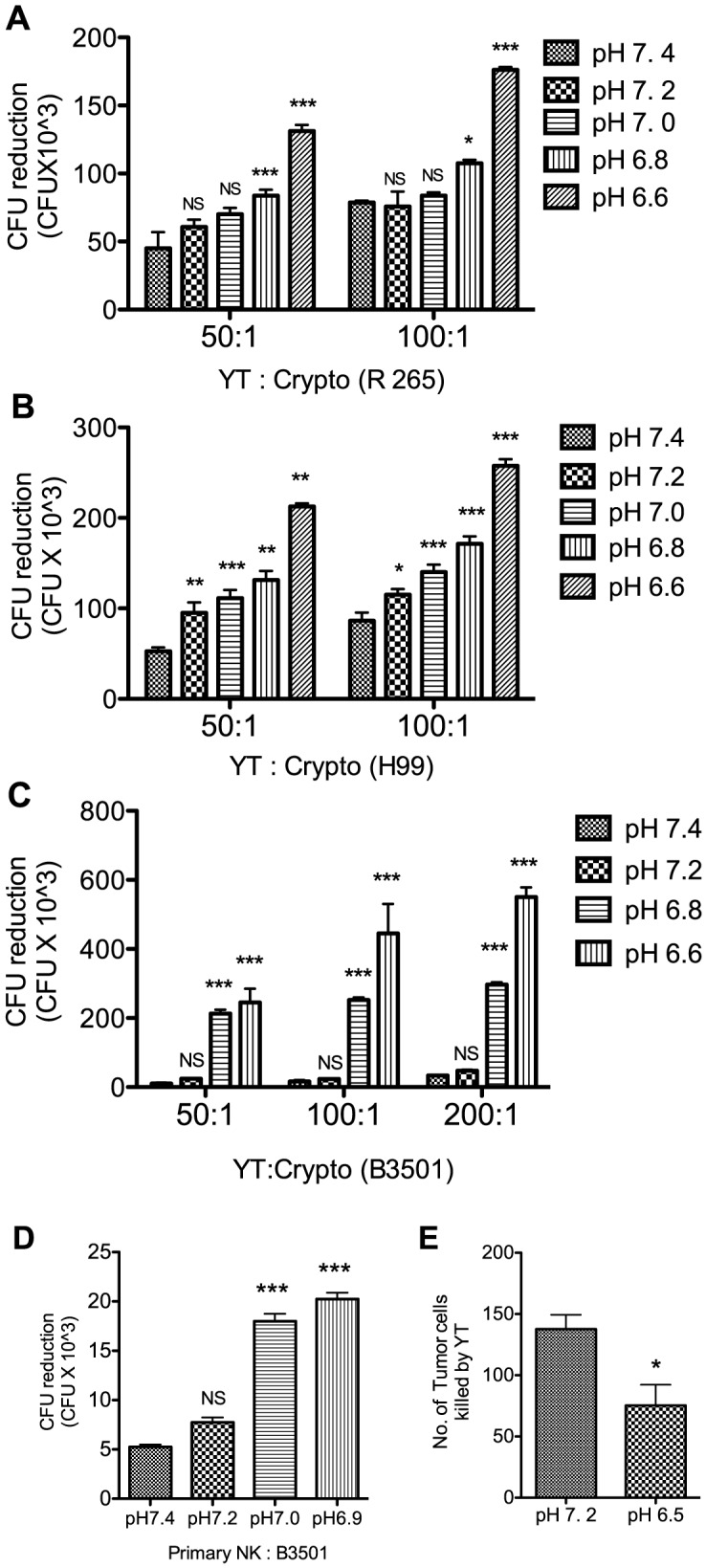
Anti-cryptococcal activity of NK cells is greater in pH 6.6 compared to pH 7.4. YT cells were co-cultured with different strains of *Cryptococcus*; *C. gattii* strain R265 (A), *C. neoformans* strain H99 (B), or *C. neoformans* strain B3501 (C). Primary human NK cells were co-cultured with B3501 (D) in different pH for 20 h. The reduction in CFU was calculated by deducting the number of experimental CFU_t = 20 h_ (*Cryptococcus* cultured with NK cells) from the number of control CFU_t = 20 h_(*Cryptococcus* cultured alone). Each bar represents the mean of four replicates. ns, not significant, *, p value≤0.05, **, p value≤0.01 ***, p value≤0.001 compared to pH 7.4. Data are representative of three (A, C and D) or two (B) experiments. (E) CFSE-labeled K562 cells (tumor cells) were cultured either alone or with YT cells at 50∶1 E∶T ratio for 4 h. The number of K562 cells killed by YT cells was calculated as described in materials and methods. Each bar represents the mean of three replicates *, p value≤0.05 compared to pH 7.2. Data are representative of three experiments.

To determine the influence of acidic pH on the direct anti-cryptococcal activity of NK cells, colony-forming units (CFU) were compared in wells with and without effector cells at various pH. The effector cells consisted of the human NK cell line, YT, and human primary NK cells isolated from PBMC of healthy volunteers. The anticryptococcal activity was assessed at different extracellular pH ranging from 7.4 to 6.6. Before each experiment the pH of the media was adjusted by adding HCl or NaOH. The pH of each well was maintained within 0.2 pH units of the starting pH. Three different strains of *Cryptococcus* were examined (*C. gattii* strain R265 and *C. neoformans* strains B3501 and H99). The anticryptococcal activity is expressed as the reduction in CFU (number of CFU_t = 20 h_ in the absence of NK cells subtracted from the number of CFU_t = 20 h_ in the presence of NK cells). Surprisingly, the YT cell-mediated reduction in the number of organisms was significantly greater in acidic pH (6.8 and 6.6) compared to physiological pH 7.4 (Figure 1A, B & C). The enhanced anti-cryptococcal activity was not strain specific as similar enhancement was observed against all three cryptococcal strains (Figure 1A, B & C). We also examined the anticryptococcal activity of primary human NK cells at acidic pH. Similar to YT cells, primary human NK cells also showed significantly greater anti-cryptococcal activity in acidic pH (7.0 and 6.9) compared to physiological pH 7.4 (Figure 1D), although survival of these cells was impaired at pH less than 6.9. These results were in contrast to previous studies by several groups reporting impaired anti-tumor activity of NK cells in acidic pH [17], [19]. Consequently, experiments were performed to confirm the antitumor activity at low pH. Consistent with previous observations, acidic pH impaired YT cell-mediated tumor cytotoxicity (Figure 1E). Thus, unlike tumor cytotoxicity that was reduced at low pH, the anticryptococcal activity of NK cells was enhanced at low pH.

### Acidic pH does not increase the effector to target ratio (E∶T)

**Figure 2:**
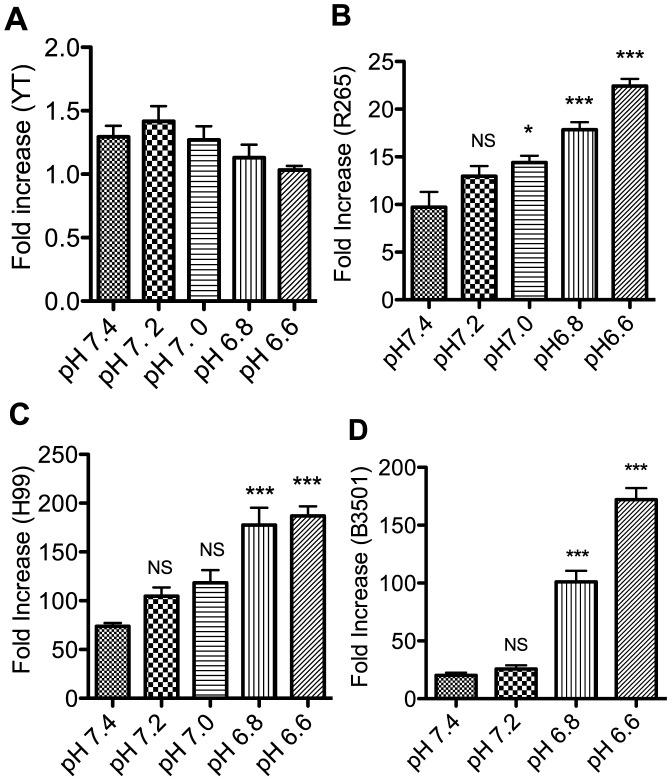
Acidic pH does not increase the effector to target (E∶T) ratio. (A) YT cells were cultured for 20 h in different pH (7.4, 7.2, 7.0, 6.8, and 6.6) and the numbers of viable YT cells were counted by light microscopy using trypan blue staining at the beginning and end of the incubation. Fold increase in the number of viable YT cells was calculated by dividing the number of viable YT cells at 20 h by the number of viable YT cells at t = 0. Each bar represents the mean of three replicates. Data are representative of three experiments. *C. gattii* strain R265 (B), *C. neoformans* strains H99 (C), or B3501 (D) were cultured in different pH (7.4, 7.2, 7.0, 6.8, 6.6) for 20 h. The number of viable organisms was detected by counting the CFU on agar plates. Each bar represents the mean of four replicates. *, p value≤0.05, ***, p value≤0.001 compared to the fold increase at pH 7.4. Data are representative of three experiments.

The unexpected observation of greater anti-cryptococcal activity in acidic pH prompted questions about the mechanism. First, we sought to determine whether the enhanced activity was due to an increase in effector cell to target (E∶T) ratio in acidic pH compared to physiological pH, which might increase killing. Increase in E∶ T ratio can occur if the number of YT cells increased or the number of *C. neoformans* decreased in acidic compared to physiological pH. We found that YT cells did not replicate faster in acidic pH as the number of YT cells increased by 1.4 fold at pH 7.4 and 1.2 fold at pH 6.6 when cultured for 20 hours (Figure 2A). On the other hand, instead of replicating at a slower rate in acidic pH compared to physiological pH, cryptococcal replication increased in acidic pH (Figure 2B, C & D). Thus, the faster replication in acidic pH resulted in a decreased E∶ T ratio (instead of increased), which should have made it more difficult for YT cells to exhibit cytotoxicity to *Cryptococcus*. Therefore, the enhanced anti-cryptococcal activities in acidic pH cannot be explained by the increase of E∶ T ratio in acidic pH compared to physiological pH.

### YT cell anti-cryptococcal activity is mediated via killing

Previous studies have demonstrated that the anticryptococcal effect of NK cells results in the absence of metabolic activity in the organisms and the final number of organisms is modestly below the starting inoculum [21]–[23]. While these studies have asserted that killing occurs as opposed to growth inhibition, we sought additional evidence that NK cells kill *Cryptococcus*. For this purpose, two different methods were used. In the first, a microwell assay for killing by NK cells was adapted from previous studies [24]. Dilutions of *Cryptococcus* in media were added to 48 wells (in a 96 well plate). According to Poisson distribution, when the dilution is achieved such that 37% of the wells are culture negative, the positive wells contain an average of one organism per well [25]. Similarly, when 10% of the wells are negative, the positive wells contain an average of 2.3 cells per well. Using this system, if NK cells kill the organisms, wells are converted from positive to negative. By contrast, if NK cells growth-inhibit the organisms, the same percentage of wells remain positive, albeit with fewer organisms. In our experiments, in the group containing *Cryptococcus* alone, 29% of the wells were negative, while in the group containing YT cells and *Cryptococcus*, 62.5% of the wells were culture negative (p<0.001 by Fisher's exact test, the experiment was performed 3 times with similar results), a statistically significant increase in the number of sterile wells, providing strong support that YT cells kill, rather than growth inhibit *C. neoformans*.

Additionally, experiments were performed to assess the ability of *Cryptococcus* to exclude propidium iodide (PI). PI is excluded by the cell membrane of viable cells but can penetrate the permeable cell membranes of dying or dead cells where it gains access to and intercalates into double-stranded nucleic acids causing the DNA to become fluorescent. There was a significant increase in the percentage of PI positive *Cryptococcus* after exposure to NK cells than in the *Cryptococcus* that had been cultured alone (43±0.8% in the presence of NK cells vs. 1±0.05% in the absence, the experiment was performed 3 times with similar results). These experiments provide additional confirmation that NK cells kill rather than growth inhibit *Cryptococcus*.

### Acidic pH does not increase the expression of active perforin within YT cells

Having previously shown that perforin is the cytotoxic molecule used by NK cells during direct cryptococcal killing [23], and that cells are stimulated to synthesize perforin during cryptococcal killing [26], we reasoned that an increase in perforin content might explain the enhanced anticryptococcal activity at low pH.

**Figure 3:**
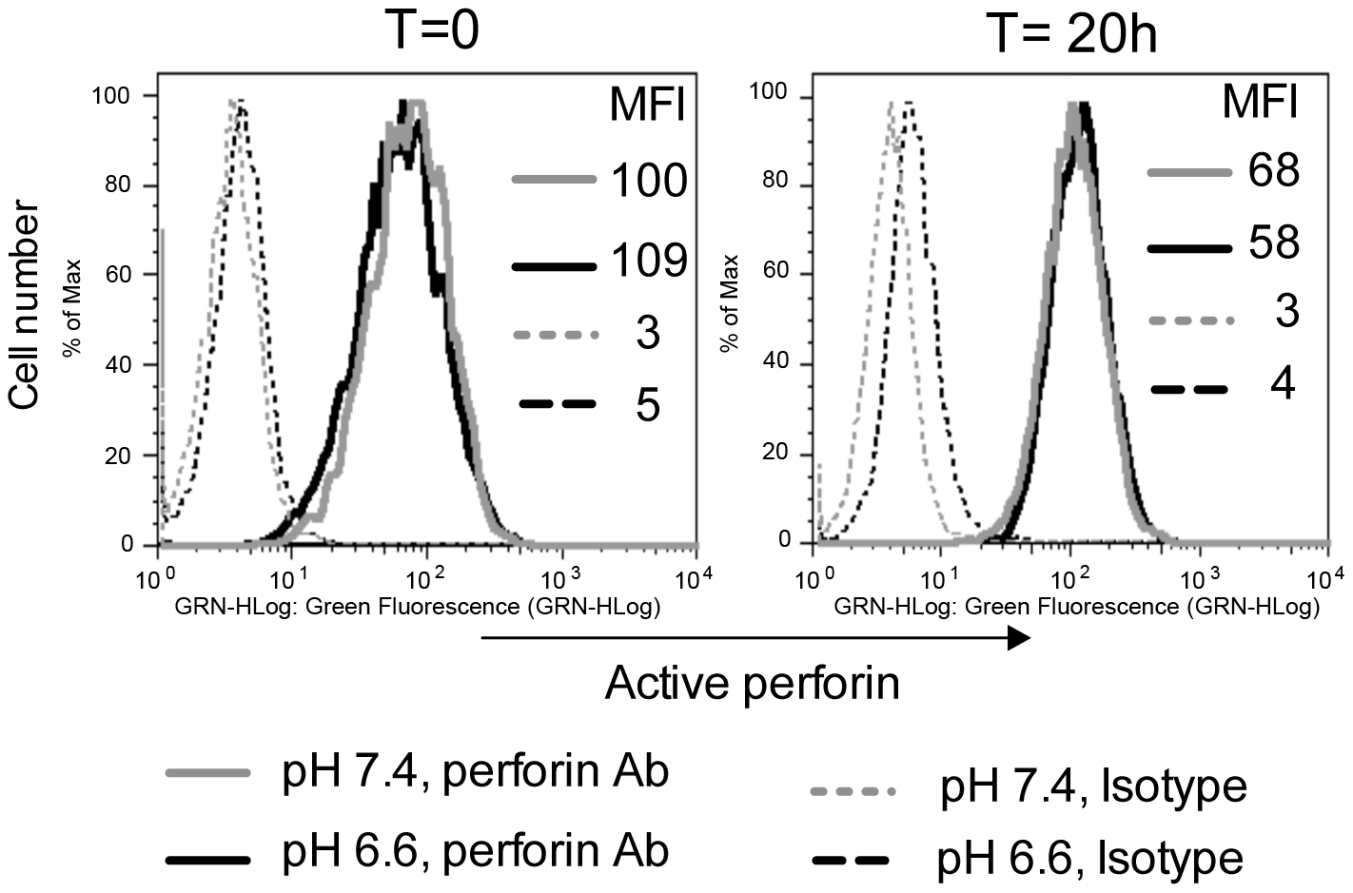
Acidic pH does not increase the amount of active perforin within YT cells. YT cells alone were cultured in complete media at pH 7.4 and 6.6 for 20 h. Cells were harvested at the beginning and end of incubation, made permeable and immunolabeled with either anti-perforin or isotype control antibodies and then analyzed by flow cytometry. Data are representative of three experiments.

Perforin is synthesized as a 70 kDa inactive precursor in the endoplasmic reticulum and transported through the Golgi complex to the acidic microenvironment of cytotoxic granules. Within these granules, the inactive precursor is cleaved to an active form by proteases that function only in acidic pH [27]. Moreover, acidic pH is also believed to favor the binding of perforin to proteoglycans, which facilitate storage [28]. Consequently, experiments were performed to determine whether an acidic extracellular pH might also increase the conversion and storage of active perforin. The expression of active perforin within YT cells was detected by a flow cytometric technique using an antibody that detected the active, 60 kDa form of perforin. The results demonstrated a similar level of active perforin in YT cells cultured in both pH 7.4 and 6.6 (Figure 3). Therefore, the increase in anticryptococcal activity is not due to an increase in active perforin in response to the acidic pH.

### Acidic pH does not increase the number of conjugates between YT cells and *C. neoformans*

**Figure 4:**
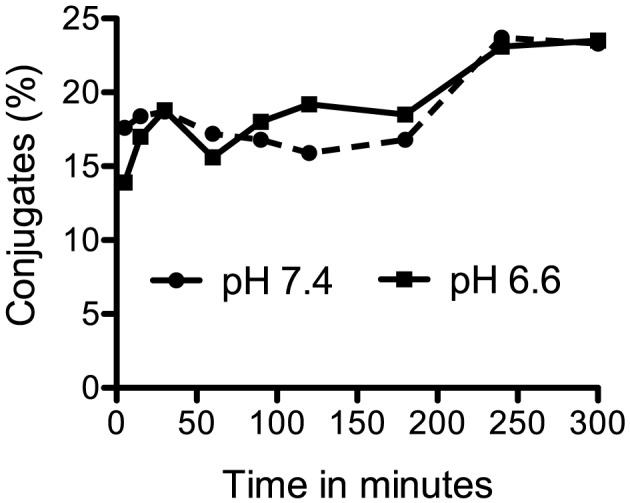
Acidic pH does not increase the number of conjugates between YT cells and *C. neoformans*. TRITC-labeled YT cells were cultured with CFSE-labeled B3501 in pH 7.4 and pH 6.6 for up to 300 minutes. At the indicated times, samples were isolated and fixed in ice-cold PBS containing 0.5% formalin and data acquired immediately using flow cytometry. Cells were identified based on their labeling and the percentage of events that simultaneously contained both TRITC-labeled YT cells and CFSE-labeled B3501 was determined. At least 5000 NK cells and 300 conjugates were counted at each time point. Data are representative of three experiments.

Having shown that there was no effect on the amount of perforin, we speculated that acidic pH might increase binding of the effector cell to the target, the first step in recognition and cytotoxicity. Although the receptor used by NK cells to recognize *Cryptococcus* has not been described, NK cells recognize pathogens directly [29] and studies of other receptors suggest that acidic pH can increase ligand binding either by increasing the affinity of receptor and ligand interaction or by increasing the expression of the receptor. For instance, neonatal Fc receptor binds with higher affinity to the Fc portion of IgG at acidic pH, and acidic pH also increases binding of FcγRII (CD32) and FcγRIII (CD16) to immune complexes [30], [31]. Moreover, acidic pH increases expression of CD18 on neutrophils, which is involved in binding to the endothelium [32]. To determine the influence of acidic pH on binding, the frequency of conjugate formation between YT cells and *C. neoformans* was assessed in physiological and acidic pH using a flow cytometric technique. YT cells (pre-labeled with TRITC) and *C. neoformans* (B3501) (pre-labeled with CFSE) were cultured together and at various times the conjugates were detected by flow cytometry. In both pH, conjugates were detectable as early as 5 minutes, which was the first time that could be assessed due to constraints of the assay. Over the 300 minutes of incubation, there was no significant increase in binding between YT cells and *C. neoformans* at pH 6.6 compared to pH 7.4 (Figure 4). These results indicate that the greater anti-cryptococcal activity in acidic pH is not due to increased conjugate formation between NK cells and *C. neoformans*.

### Acidic pH increases ERK1/2 signaling within YT cell upon cryptococcal stimulation

**Figure 5:**
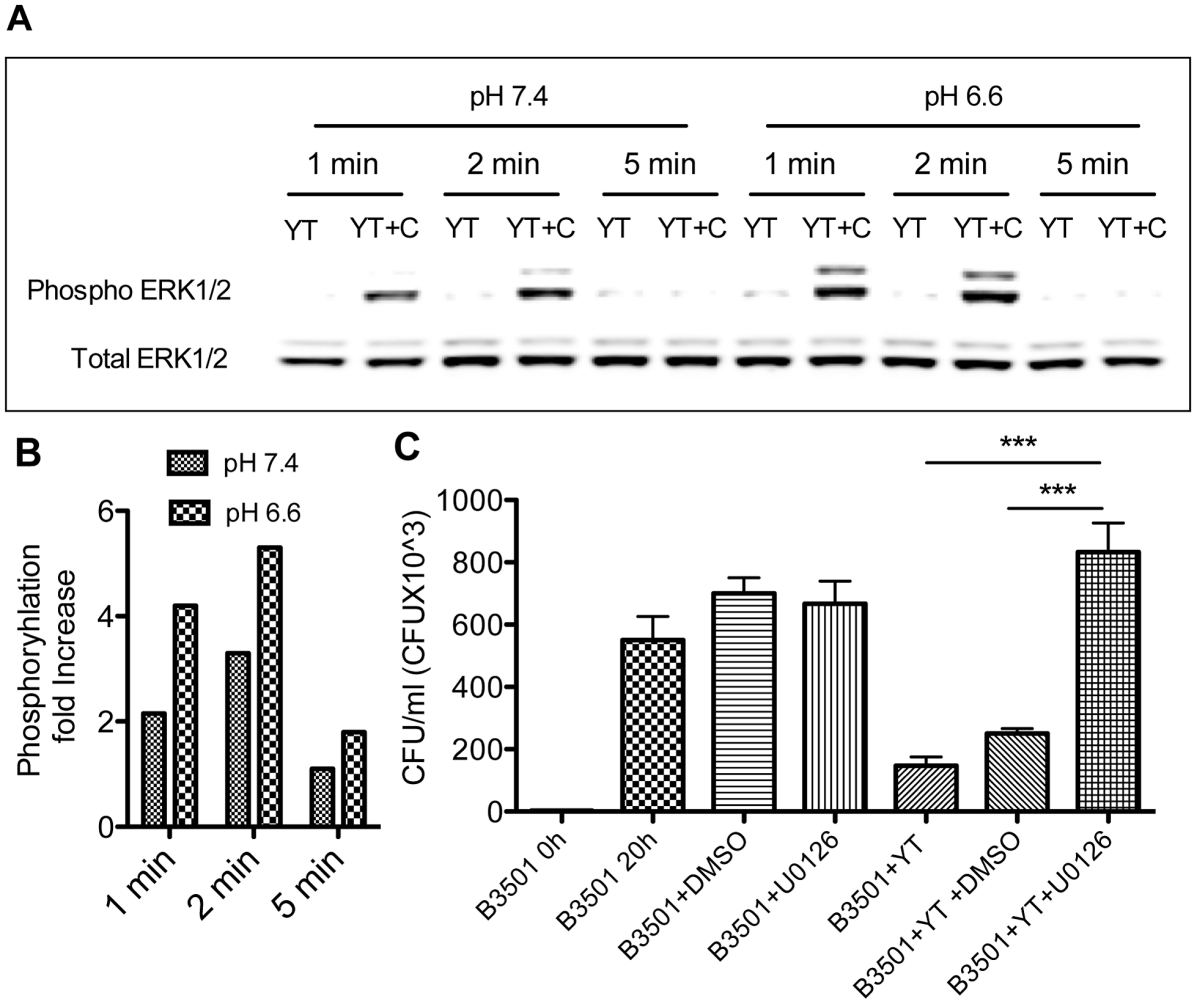
*Cryptococcus*-induced ERK1/2 signaling in YT cells is greater in pH 6.6 compared to pH 7.4. (A) YT cells were left unstimulated (YT) or stimulated with B3501 (YT+C) at E∶T ratio of 1∶100 at 1, 2 and 5 minutes in pH 7.4 and 6.6. Immunoblotting was performed to detect dual Tyr/Thr phosphorylation of ERK1/2 (p-ERK1/2). Total ERK1/2 was used as a loading control. Data are representative of three experiments. YT, YT cells; C, *Cryptococcus*. (B) Densitometry of the bands in A was performed using LI-COR Odyssey instrument software and the fold increase in ERK1/2 phosphorylation was calculated by dividing the density of the stimulated bands with the density of unstimulated bands at each time point. Data are representative of three experiments. C. YT cells were pretreated for 2 h with 50 µM U0126 and anticryptococcal activity was compared with untreated and vehicle control (DMSO). ***, p<0.0001. Data are representative of three experiments.

Following receptor and ligand binding, intracellular signaling is induced, ultimately leading to the process of cytotoxicity. This provides a second opportunity for signal amplification following receptor binding. NK cell cryptococcal killing requires PI3K-dependent ERK1/2 signaling [21]. In particular, the PI3K-ERK1/2 pathway is required for polarization of perforin-containing granules and the release of perforin following conjugate formation. To compare ERK1/2 activation in acidic vs physiological pH, YT cells were stimulated with *C. neoformans* over a time course and immunoblots for dual tyrosine/threonine phosphorylation of p42/44 MAPK (ERK 1/2) was performed. At both pH 7.4 and pH 6.6, the phosphorylation of ERK1/2 was detected as early as 1 min, which reached a maximum at 2 minutes, and returned toward baseline at 5 minutes. Although the time course was similar in both pH, the intensity of phosphorylation of both ERK1 and ERK2 was substantially higher at both time points (1 min and 2 min) in pH 6.6 compared to pH 7.4 (Figure 5A and B). These results indicate that the enhanced anticryptococcal activity was accompanied by increased ERK 1/2 signaling.

Although *Cryptococcus* induced ERK1/2 signaling was greater at pH 6.6 compared to pH 7.4, it was not known whether ERK1/2 activation was required for cryptococcal killing in pH 6.6 as it is at pH 7.4 [21]. To address this question, YT cells were pretreated with U0126, a specific inhibitor of MEK1 and MEK2, which are the immediately upstream of ERK1 and ERK2. YT cells treated with U0126 were significantly (p<0.0001) impaired in their ability to kill *C. neoformans* at pH 6.6 compared to vehicle control (DMSO)-treated YT cells (Figure 5C). The viability of YT cells was not altered with these treatments.

### *C. neoformans* induces greater perforin degranulation in pH 6.6 compared to pH 7.4

**Figure 6:**
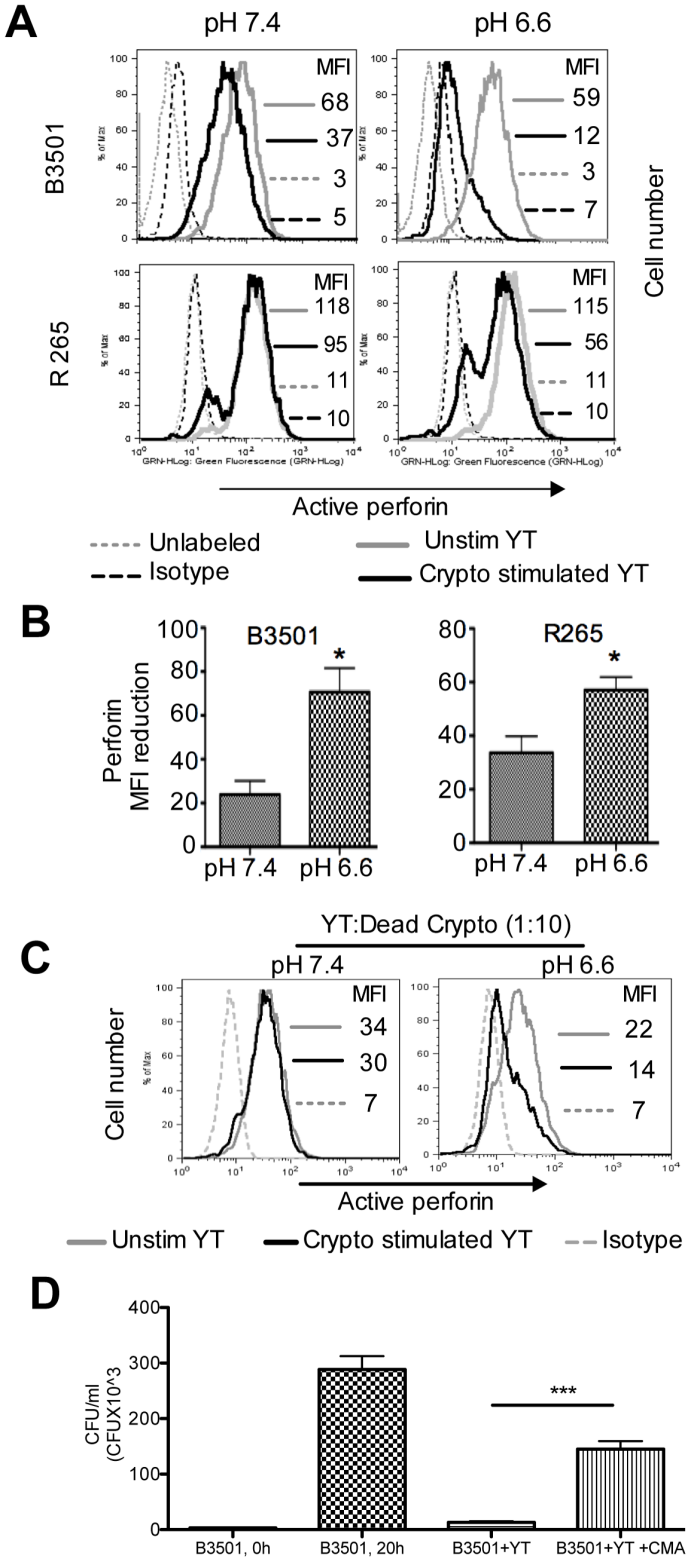
*Cryptococcus*-induced perforin loss in YT cells is greater in pH 6.6 compared to pH 7.4. (A) YT cells were cultured alone or with live *Cryptococcus* (B3501 in upper panel and R265 in lower panel, E∶T, 1∶10) in pH 7.4 and 6.6 for 20 hours. Cells were harvested, made permeable and immunolabeled with either anti-perforin or isotype control antibodies. Data are representative of three experiments. (B) Reduction in perforin MFI was calculated by deducting the MFI of *Cryptococcus* stimulated YT from the MFI of unstimulated YT. (C) Same as A except for heat killed B3501. Data are representative of three experiments. (D) YT cells were pretreated for 2 h with 10 nM concanamycin A (B3501+YT+CMA) and anticryptococcal activity was compared with untreated group (B3501+YT). ***, p<0.0001. Data are representative of three experiments.

Perforin degranulation is required for cryptococcal killing [21]. As acidic pH was associated with increased signaling, experiments were performed to determine whether this increased signaling translated into enhanced degranulation at acidic pH as assessed by measuring the loss of active perforin after stimulation with live *C. neoformans* (B3501) and *C. gattii* (R265). Interestingly, there was a more pronounced loss of active perforin in pH 6.6 compared to pH 7.4 (Figure 6A & B).

While the most likely explanation for the loss of perforin was due to degranulation, there are other possibilities. To confirm degranulation, expression of lysosome associated membrane protein-1 (LAMP-1, CD107a) was assessed. In resting cells, LAMP-1 is present within the granule membrane but absent from the cell surface. When granules fuse with the extracellular membrane during degranulation, LAMP-1 is exposed on the surface of cytotoxic cells, allowing the anti-LAMP-1 antibody to bind. YT cells were co-cultured with *C. neoformans* (strain B3501) for 18 h in the presence of FITC-conjugated anti-LAMP-1 antibody. At pH 6.6 the percentage of LAMP-1 positive cells increased 3-fold compared to a 2.6 fold increase at pH 7.4 consistent with increased degranulation at low pH.

We considered the possibility that in addition to the low pH, the number of organisms that are available to stimulate the NK cells might also affect degranulation. Although the same number of *Cryptococcus* were added at the beginning of the assay, the faster replication in acidic pH results in more organisms at pH 6.6 compared to pH 7.4 throughout much of the experiment. Thus, experiments were performed to separate the influence of pH from the larger number of organisms. For this purpose, experiments were performed using the same number of dead *C. neoformans* at different pH. When YT cells were stimulated with dead *Cryptococcus* at an effector to target ratio of 1∶10, there was modest loss of perforin in pH 7.4 after 20 h of incubation, similar to our previous observations [26]. However, at pH 6.6, the same number of dead organisms caused substantially greater loss of perforin (Figure 6C). These data indicate that the acidic pH by itself can enhance the *Cryptococcus* induced degranulation in YT cells, independent of the number of organisms.

Although killing of *Cryptococcus* was enhanced in pH 6.6 and perforin degranulation was greater in pH 6.6 compared to pH 7.4, it was not known whether perforin was required for cryptococcal killing at pH 6.6. Concanamycin A is an inhibitor of the vacuolar ATPase that is required to maintain active perforin. Previous studies have shown that concanamycin A selectively inhibit perforin-mediated NK cell cytotoxicity on *Cryptococcus* at pH 7.4 [23]. YT cells treated with concanamycin A were significantly (p<0.0001) impaired in their ability to kill *C. neoformans* at pH 6.6 (Fig. 6D). These data suggest that, similar to pH 7.4, the perforin-mediated cytotoxic pathway is important for the anticryptococcal activity of YT cells at pH 6.6. The viability of YT cells was not altered by treatment with concanamycin A.

### NK cell killing is proportional to cryptococcal replication

**Figure 7:**
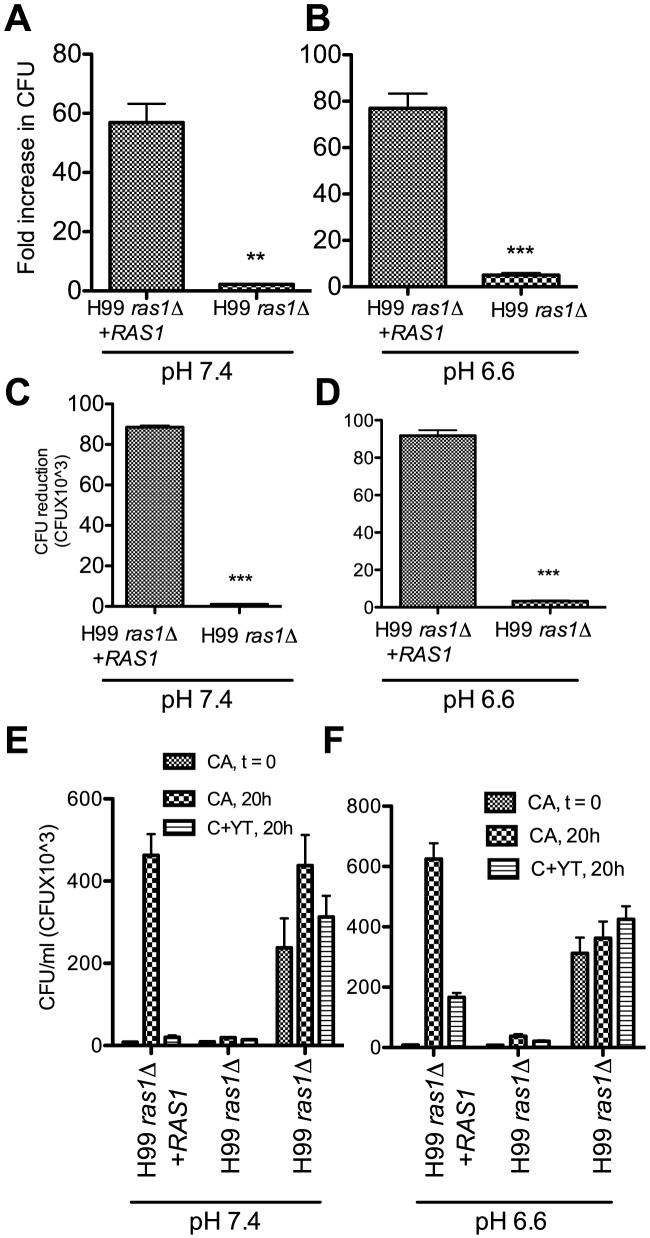
NK cell mediated cryptococcal killing is proportional to the rate of replication of the organisms. (A&B) H99*ras1Δ* and H99*ras1Δ*+*RAS1* were cultured alone at 37°C for 20 h in pH 7.4 (A) and pH 6.6 (B). Fold increase in CFU was determined by dividing the CFU_t = 20 h_ by CFU_t = 0_. (C&D) H99*ras1Δ* and H99*ras1Δ*+*RAS1* were cultured with or without YT cells at 37°C for 20 h in pH 7.4 (C) and pH 6.6 (D). The CFU reduction was calculated as described in figure 1. Each bar represents the mean of four replicates. Data are representative of three experiments. (E&F) H99*ras1Δ* and H99*ras1Δ*+*RAS1* were either cultured alone or with YT cells at 37°C for 20 h at pH 7.4 (E) and pH 6.6 (F) and the CFUs for each time point (t = 0 and t = 20 h) are shown. For H99*ras1Δ*, two different starting inocula are shown. Each bar represents the mean of four replicates. Data are representative of two experiments.

Although low pH increased degranulation, these results do not exclude the possibility that the faster replication might also contribute to greater degranulation. To evaluate whether cryptococcal replication influences NK cell killing, a temperature-dependent strain of H99 was employed. The *ras1* knockout strain of H99 (H99*ras1*Δ) replicates slowly at 37°C, while the *ras1* reconstituted strain (H99*ras1*Δ+*RAS1*) replicates at a faster rate. These studies were made possible because low pH has a modest effect on the replication of H99*ras1*Δ and H99*ras1*Δ+*RAS1*. Studies were performed to determine whether killing of H99*ras1*Δ and H99*ras1*Δ+*RAS1* were dependent on the replication independent of pH. At pH 7.4 and 37°C, H99*ras1*Δ+*RAS1* replicated 57 fold, while H99*ras1*Δ replicated only 2 fold within 20 hours of culture (Figure 7A). Under these conditions, YT cells killed a significantly higher number of H99*ras1Δ*+*RAS1* compared to H99*ras1Δ* (Figure 7C). Similarly, at pH 6.6 and 37°C, H99*ras1Δ*+*RAS1* replicated 76 fold, but H99*ras1Δ* replicated only 5 fold (Figure 7B), and YT cells killed significantly higher number of H99*ras1Δ*+*RAS1* compared to H99*ras1Δ* (Figure 7D). Thus, NK cell killing was proportional to the cryptococcal replication independent of the pH, providing an additional explanation for enhanced susceptibility to NK microbicidal activity.

We considered the possibility that the slower replicating H99*ras1Δ* would present fewer available targets, which might explain the reduced killing. To answer this question, the killing assay was repeated using high and low starting inocula of H99*ras1Δ*. The results showed that despite a higher starting inoculum and similar numbers of organisms at 20 hours, YT cells killed H99*ras1Δ*+*RAS1* to a greater extent when compared to H99*ras1Δ* independent of the number of organisms. Similar results were observed at both pH 7.4 and 6.6 (Figure 7E & F). Thus, the NK cell killing is proportional to the replication of the organism independent of the number of organisms.

### NK cells are present within a cryptococcoma

**Figure 8:**
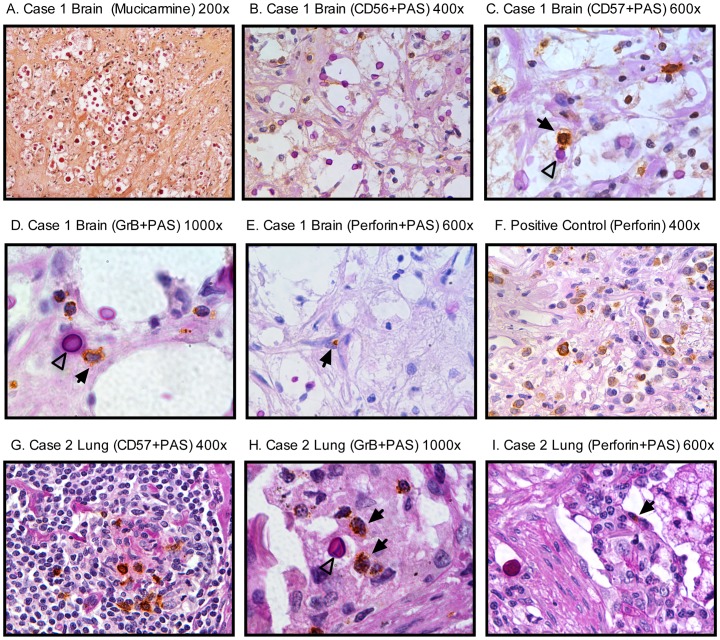
Cryptococcomas contain numerous granzyme B positive, but very few perforin positive NK cells. Case 1: The brain was removed at autopsy and fixed in formalin with subsequent sectioning and examination. Sections were taken from the cryptococcoma in the cerebral cortex and stained with mucicarmine (200×) (A); or stained with PAS and labeled with either anti-CD56 (400×) (B); anti-CD57 (600×) (C); anti-granzyme B (1000×) (D); or anti-perforin (E, 600×) antibody. *Cryptococcus* (open arrow) in association with labeled cells (solid arrows) are shown. Perforin positive cells (brown) in NK cell lymphoma in lung, shown as a positive control for perforin (F). Case 2: Wedge resection of lung cryptococcoma, fixed in formalin with subsequent sectioning, PAS staining and labeled with either anti-CD57 (G, 400×), anti-granzyme B (H, 1000×) or anti-perforin (I, 600×). No organisms are present in figure G. The organisms stain bright pink with PAS, whilst positive staining for antigens is dark brown.

Having observed that an acidic pH enhanced NK cell cryptococcal killing, we sought to determine whether NK cells with cytolytic potential are present within a persistent cryptococcoma. Histopathological examination of autopsy sections taken from the cerebral cortex of a patient who acquired *C. gattii* in northwestern Washington State revealed numerous focal collections of mucicarmine positive fungal organisms with surrounding inflammatory cells (figure 8A), which is typical of a cerebral cryptococcoma. NK cells were identified by the presence of numerous CD56+ (481 CD56+ cells/10 HFP, figure 8B) and CD57+ (425 CD57+ cells/10 HFP figure 8C) cells within the cryptococcoma. Some of these cells (solid arrowheads) could be found intimately associated with *Cryptococcus* (open arrowhead). Many cells were granzyme B positive (500 GrB+ cells/10 HFP figure 8D) suggesting cytotoxic potential, but very few were perforin positive (1 perforin+ cells/10 HFP figure 8E) A right lower lobe lung nodule that was suspicious for cancer was resected from an asymptomatic immunocompetent patient who was exposed to *Cryptococcus* on Vancouver Island, Canada. The nodule contained numerous mucicarmine positive organisms (not shown) consistent with cryptococcoma (although the species was not determined, the clinical presentation would be most consistent with *C. gattii*). CD56+ (not shown) and CD57+ cells (figure 8G) were present within the lesion. Similar to the first case, granzyme B positive cells were present (figure 8H), but a paucity of perforin positive cells (figure 8I). These cases suggest that cells within cryptococcoma have cytotoxic potential but are depleted of the critical effector molecule, perforin, or not recruited.

## Discussion

In this study, we made the following observations. 1) Anti-cryptococcal activity of YT cells and primary NK cells is enhanced in acidic pH, which is in contrast to their decreased antitumor activity. 2) Anti-cryptococcal activity of YT cells is mediated via killing. 3) Acidic pH does not increase conjugates with *Cryptococcus* or the active perforin content of YT cells. 4) Acidic pH enhances the *Cryptococcus*-induced ERK1/2 signaling as well as perforin degranulation in YT cells, suggesting that these are the mechanisms of greater killing in acidic pH. 5) Acidic pH enhances the cryptococcal replication, and within the same pH, YT cells kill the faster replicating *Cryptococcus* more efficiently than the slower replicating organisms, indicating that faster cryptococcal replication also plays a role in greater killing in acidic pH. 6) NK cells were found within a cerebral and a lung cryptococcoma. While numerous granzyme B positive cells were found intimately associated with the organism, very few perforin positive cells were identified within the cryptococcoma.

Previous studies reported direct influences of acidic extracellular pH on a variety of immunological functions, and in particular, acidic pH was reported to impair the direct cytotoxic activity of NK cells on tumor cells [17], [33]. Here, we investigated the influence of acidic extracellular pH on the direct cytotoxic activity of NK cells on a fungal cell, *Cryptococcus*. At physiological pH, similar to tumor cells, NK cells are known to directly bind and exhibit cytotoxicity to *Cryptococcus* [34]. Moreover, NK cells use perforin as a cytotoxic molecule for both *Cryptococcus* and tumor cells [23]. During antitumor and anticryptococcal cytotoxicity, NK cells share the following stepwise mechanisms: binding with the target, PI3K-dependent ERK1/2 signaling and reorientation of the perforin-containing granules towards the synapse followed by degranulation [21]. Considering these similarities, we hypothesized that similar to the antitumor activity, the anticryptococcal activity of NK cells would be impaired in acidic extracellular pH. Surprisingly, we found that both human primary NK cells and the human NK cell line, YT, exhibit greater anti-cryptococcal activity in acidic compared to physiological pH. This acidic pH-mediated enhancement of anticryptococcal activity appears to be a general phenomenon because we observed similar enhancement of NK cell activity against different species and strains of *Cryptococcus* (R265, H99 and B3501). By contrast, we found that YT cells killed less tumor cells (K562, a NK cell sensitive tumor cell line) in acidic compared to physiological pH, which is consistent with previous reports. These results indicate that the acidic pH has the capability to enhance the NK cell cytotoxicity against a microbial cell, but to impair the same function against a tumor cell despite the similarities between these two mechanisms.

It is possible that fundamental differences between the structures and genetic make-up of *Cryptococcus* and tumor are responsible for these divergent outcomes. Indeed, previous studies also reported differences between the mechanisms of antitumor and anticryptococcal activity of NK cells. For example, NK cells take more time to exhibit a cytotoxic effect on *Cryptococcus* compared to tumor cells [35], [36]. While NK cells require LFA-1 for binding and killing of tumor cells, the anticryptococcal activities occur in the absence of LFA-1 [37]. NK cells bind to *Cryptococcus* through many microvilli that penetrate the capsular material to associate with the cryptococcal cell wall, which is in contrast to the broad membrane-membrane interactions that occurs during binding to tumor cells [34].

The current studies extend our knowledge of the differences between tumoricidal and microbicidal cytotoxicity. Previous studies also reported acidic pH-mediated enhancement of different immune cell function. For instance acidic pH was reported to delay neutrophil apoptosis, extend neutrophil functional lifespan as well as to increase the surface expression of CD18 on neutrophils [32]. Acidic pH was reported to markedly increase the expression of HLA-DR, CD40, CD80, CD86, CD83, and CCR7, and to improve the T cell priming ability of dendritic cells. The IL-12 production by dendritic cells was also increased in acidic pH [38]. Acidic pH was reported to activate the alternate pathway of complement [39]. Thus, our study provides new information about immune responses that are enhanced by acidic conditions, which are often found in inflammation, tumor and microbial infections.

Recognition and binding of *Cryptococcus* is the first step during NK cell cryptococcal killing, which is believed to occur through the interactions between receptor and ligand [40]. Unfortunately, the identity of the receptor involved during binding has not been described. Nevertheless, prior studies indicate that the acidic microenvironment can enhance the binding between immune cells and their targets. Acidic extracellular pH was reported to enhance the binding of neutrophils with endothelium as well as the binding of human melanoma cells with the extracellular matrix [41], [42]. The integrins are heterodimeric α/β transmembrane receptors that are expressed by leukocytes and facilitate both adherence to the vascular endothelium and other cell-to-cell interactions. Some integrins must be activated before they can bind with high affinity to their ligands and acidic extracellular pH was reported to increase the level of activated α_v_β_3_ integrin on the cell surface, resulting in increased binding with the target [43]. However, our studies failed to demonstrate a difference in binding at any time over a 5-hour period of co-culture. These data suggest that greater killing in acidic pH does not occur due to increased binding in acidic conditions.

During cryptococcal killing, PI3K is activated in both primary human NK and YT cells, and this activation is essential for YT cell-mediated cryptococcal killing. Downstream of PI3K, ERK1/2 is activated in a PI3K-dependent fashion and is required for cryptococcal killing [21]. It is not clear how the acidic pH enhances ERK1/2 signaling during cryptococcal killing, but there are several possibilities. Evidence suggests that the acidic pH may enhance other types of signaling without increasing the frequency of binding. For example, the G protein coupled receptor, β_2_AR signals by activating the G protein, Gs. The basal (agonist-independent) activation of Gs by the β_2_AR is greater at pH 6.5 compared with pH 8.0 indicating enhanced signaling at low pH [44]. Alternatively, acidic pH can also activate MAPK signaling in different cells. For instance, acidic pH (pH 6.5) was reported to induce neutrophil and dendritic cell activation through the activation of the ERK1/2 MAPK pathway [38], [45]. One study reported acidic pH (pH 6.6) induced activation of the ERK1/2 MAPK pathway in human glioblastoma cells, which induced vascular endothelial growth factor (VEGF) [46]. Additional studies have shown that extracellular protons can be recognized by a subfamily of G protein-coupled receptors, which includes four receptors: G protein-coupled receptor 4 (GPR4), ovarian cancer G protein-coupled receptor 1 (OGR1), T cell death-associated gene 8 (TDAG8), and G2 accumulation (G2A). Although these receptors were originally characterized by their ability to bind inflammatory lipids, studies reported that extracellular protons could activate all of these receptors inducing a variety of signaling pathways including the MAPK pathway [47]–[49]. Moreover, TDAG8 has been reported to be expressed in human NK cells, suggesting that NK cells are able to sense extracellular protons [50].

We considered the possibility that in addition to the direct effect of pH, replication of organisms might independently enhance the microbicidal activity. We have examined this issue by employing a temperature sensitive mutant of *C. neoformans* that replicates more slowly at 37°C than the reconstituted strain at the same pH [51]. Cryptococcal killing was directly proportional to the replication of this organism as YT cells were able to kill a greater number of faster replicating *C. neoformans* compared to the slower replicating ones even when a large number of slower growing organisms were present. These results indicate that the faster cryptococcal replication also has an important role in the enhancement of NK cell killing in acidic conditions. We do not know what is making the faster replicating *Cryptococcus* more susceptible to NK cell killing. However, two possibilities come to mind. *Cryptococcus* might be susceptible to cytolytic molecules only during certain stages of the cell cycle. Specifically, the *Cryptococcus* may be susceptible in one of the active stages of the cell cycle (G1, S, G2 and M) rather than the resting phase (Go) [52]. As cells progress through the cell cycle, they undergo changes in membrane structure and function [53], and susceptibility to various drugs [54]. In fact, there is evidence in the literature demonstrating the influence of target cell cycle on direct immune cell-mediated killing. It has been reported that the position of a malignant cell in the cell cycle affected their susceptibility to cytotoxic T lymphocyte (CTL)-mediated lysis [55] as well as to antibody plus complement-mediated lysis [56]. During replication, *C. neoformans* also goes through certain cell cycle-specific changes in their structure that have high potential to influence the outcome of direct NK cell mediated killing. Alternately, changes in surface structure during the cell cycle might alter recognition rather than susceptibility. Cells going through the cell cycle undergo changes in expression of surface antigens [57]. Using tumor and virus infected tissue targets, studies showed that NK cells react more efficiently to the proliferating cells than the non-proliferating ones [58]. These structural variations during different stages of cell cycle indicate that the ligand that is recognized by NK cell receptors might also be expressed variably during certain phases of cell cycle. As a result, during faster replication, *Cryptococcus* goes through cell cycle more frequently, yielding more organisms that are recognized for NK cell killing.

As the acidic pH enhances the ability of NK cells to kill *Cryptococcus*, the current studies would predict that the acidic microenvironment is not responsible for the failure to eliminate a cryptococcoma. However, persistence of cryptococcoma indicates that there must be other reasons that NK cells fail to eradicate this fungus. While very low pH (pH<6.6), might disrupt cellular physiologic processes and cytotoxicity, to understand the failure to clear cryptococcoma, we investigated the relationship of NK cells to the organisms within the cryptococcoma. Histopathological examination demonstrated the presence of NK cells within the cryptococcoma. Although these cells were strongly positive for granzyme B, suggesting that these NK cells possess cytotoxic potential, very few perforin positive cells were present suggesting that the cells within the persistent cryptococcoma were depleted of perforin, the critical effector molecule, or were not recruited. This observation may explain why the NK cells fail to eradicate the cryptococcoma despite their enhanced ability to kill *Cryptococcus* in acidic microenvironment. We acknowledge these studies are limited because samples were obtained from only two patients, but they may provide insights that could translate to therapies. It may be possible to provide the signals necessary to activate NK cells to express perforin, or to provide the chemotactic signals that would recruit microbicidal NK cells into the cryptococcoma.

In conclusion, we have identified acidic pH-mediated enhancement of direct microbicidal activity of NK cells, which is in contrast to their impaired tumoricidal activity. The increased killing correlates with acidic pH mediated enhancement of ERK1/2 signaling, perforin degranulation and cryptococcal replication.

## Materials and Methods

### Preparation of *Cryptococcus*

*C. neoformans* strain H99 (encapsulated, serotype A) and B3501 (encapsulated, serotype D) were obtained from ATCC (American Type Culture Collection) (Manassas, VA, USA). *C. gattii* strain R265, (encapsulated, serotype B) was obtained from Jim Kronstad of University of British Columbia,Vancouver, Canada. The temperature-sensitive mutant H99*ras1Δ*, H99*ras1Δ*+*RAS1* were obtained from J. Andrew Alspaugh, Duke University Medical Center, Durham, North Carolina, USA [51]. All of these strains were maintained on Sabouraud dextrose agar slants (BD Biosciences, Mississauga, Canada) as previously described [59]. The organism was transferred from slants to Sabouraud dextrose broth (BD Biosciences, Mississauga, Canada) and placed onto an orbital shaker at 32°C for 48 hours and then stored at 4°C to use as a stock culture. *Cryptococcus* was grown to log phase in Sabouraud dextrose broth (BD Biosciences, Mississauga, Canada) at 32°C with gentle rotation in an orbital shaker for 24 hours before experiments.

### Antibodies and reagents

The following reagents were used: FITC-conjugated anti-perforin antibody (clone δG9), anti-CD11a-PeCy5, anti-CD3-PE, anti-CD56-FITC, anti-CD14-PE and, anti-CD19-PE (all from BD Biosciences, Mississauga, Canada); red fluorescent dye TRITC (Sigma-Aldrich, St. Louis, MO); carboxyfluorescein di-acetate succinimidyl ester (CFSE) (Guava Technologies, Hayward, CA, USA). Anti-phospho-ERK1/2 (p-ERK1/2) and, anti-total-ERK antibodies were obtained from Cell Signaling Technology, Inc. (Danvers, MA, USA). The secondary antibodies (IR700 anti-rabbit and IR800 anti-mouse) for visualization of immunoblots using the Odyssey infrared imager (LI-COR Biosciences) were obtained from LI-COR Biosciences, Lincoln, Nebraska USA. Antibodies used for immunohistochemistry were anti-CD56 (Life Technologies, Burlington, ON), anti-CD57 (Cell Marque, Rocklin, CA), Granzyme B (Dako, Burlington, ON) and anti-perforin (Acris Antibodies, San Diego, CA).

### Cell lines and primary human NK cells

YT cells (human NK cell-like thymic leukemia cells) were obtained from Dr. C. Clayberger (NIH, Bethesda, MA) and stored in liquid nitrogen until they were used. Cells were cultured in complete media consisting of RPMI 1640 (with HEPES buffer), 10% heat inactivated fetal bovine serum (Invitrogen Life Technologies, Burlington, Canada), 2 mM L-glutamine, 1 mM sodium pyruvate and 0.1 mM non-essential amino acids, 100 U/ml penicillin and 100 µg/ml streptomycin (all from Invitrogen Life Technologies, Burlington, Canada). Cells were passaged every 3 to 4 days and experiments were performed using cells between passage 5–20.

Human primary NK cells were isolated from peripheral blood mononuclear cells (PBMC) as described previously [26]. Briefly, peripheral blood was obtained by venipuncture from healthy volunteers who had no history of cryptococcosis after obtaining signed consent (protocol approved by the Conjoint Health Research Ethics Board of the University of Calgary). PBMC were isolated by centrifugation at 650×g for 20 minutes (Sorvall Legend RT; Sorvall Heraeus) over a ficoll-hypaque density gradient (Sigma-Aldrich, St. Louis, MO). PBMC were harvested and washed three times in Hanks' balanced salt solution (Invitrogen Life Technologies, Burlington, Canada). The NK cells were isolated from PBMC by the MACS negative selection system on the AutoMACS cell sorter using the NK cell isolation kit (both from Miltenyi Biotec, Bergisch Gladbach, Germany). The purity of NK cells was determined by flow cytometry using anti-CD3-PE, anti-CD56-PE, anti-CD14-PE, anti-CD19-PE and, anti-CD16-FITC antibodies (all from BD Biosciences, Mississauga, Canada). NK cells were cultured overnight in complete media as described above. K562 cells (erythroleukemia cells) were a gift from Oliver Bathe (University of Calgary, Calgary, Canada) and maintained in complete medium.

For the some experiments, YT cells were pretreated with 50 µM U0126 (Calbiochem, La Jolla, CA) for 2 h before culturing with *Cryptococcus*. In other experiments, YT cells were pretreated with 10 nM concanamycin A (Sigma-Aldrich) for 2 h. After 2 h incubation, cells were washed three times with the media before culturing with *Cryptococcus*. The viability of YT cells was not altered by these treatments as assessed by trypan blue exclusion.

### Anticryptococcal activity

Anticryptococcal activity was assessed by determining the number of colony forming unit (CFU) as previously described by Mody et al [59]. In brief, *Cryptococcus* (approximately1×10^3^ cells/well) was cultured alone or with YT cells (starting E∶T = 50∶1, 100∶1, 200∶1) or with primary NK cells (starting E∶T = 800∶1) for 20 hours in 37°C and 5% CO2. The number of CFU was determined at 0 and 20 hours by lysing the effector cells in water followed by diluting and spreading onto Sabouraud dextrose agar plates. Results are expressed as the reduction in the number of *Cryptococcus* according to the following formula (Reduction in number of *Cryptococcus* = CFU control_20 h_ - CFU experimental_20 h_). Before each experiment, the media (RPMI 1640 with HEPES buffer) was adjusted to different pH (pH 7.4, 7.2, 7.0, 6.8, 6.6) using HCl or NaOH and monitored during the experiment. Measurement of pH at the end of experiments revealed that the pH was maintained within 0.2 units of the starting pH.

### Antitumor activity

NK cell anti-tumor activity was measured using the Guava EasyCyte CellToxicity Kit (Guava Technologies, Hayward, CA, USA) following the manufacturer's protocol. Briefly, the tumor cell target K562 was pre-labeled with 2.5 µM CFSE and cultured in triplicate at 37°C and 5% CO_2_ for 4 h with NK cells at 50∶1 E∶T ratio, following which 20 µl of 7-AAD [a dye that labels dead cells [60]] was added to each well and the cells incubated for a further 10 min at room temperature in the dark. Samples were acquired on the Guava EasyCyte flow cytometer (Guava Technologies, Hayward, CA, USA) and analyzed using the Guava CellToxicity software (Tree Star, Ashland, OR, USA). K562 cells were identified by CFSE staining and the number of K562 cells killed by YT cell was calculated by deducting the number of 7-AAD positive K562 that were cultured alone (spontaneously dead) from the number of 7-AAD positive K562 that were cultured with YT cells.

### Limiting dilution-based microwell assay

An assay of cryptococcal killing was modified from the technique of Schaffner et al [24]. Dilutions of *Cryptococcus* in media were added to 96 wells with the goal of achieving 10–37% of wells culture negative. YT cells were added to half of the well to achieve confluence to encourage contact and cultured at 37°C and 5% CO2 for 48 hours. To determine whether wells contained viable organisms (positive), dH_2_O was added to each well of the groups with or without YT cells and the entire content of each well was cultured on agar plates to assess the presence of one or more viable organisms.

### Propidium Iodide assay

CFSE-labeled B3501 was co-cultured with YT cells for 20 h. At the end of incubation, PI (propidium iodide) was added to the culture, B3501 was identified based on CFSE labeling and the PI positive B3501 was detected by flow cytometry.

### Conjugate formation assay

Conjugate formation between YT cells and *Cryptococcus* was performed as previously described [37] with some modifications. Briefly, *Cryptococcus* was pre-labeled with the green fluorescent dye CFSE, while YT cells were pre-labeled with the red fluorescent dye TRITC, following the manufacturer's protocol (Sigma-Aldrich, Ontario, Canada). *Cryptococcus* (5×10^5^) were mixed with YT cells (1×10^5^) in 200 µl final volume of cold complete medium, centrifuged at 27 g for 5 minutes and incubated at 37°C for the indicated time periods. Cells were then fixed in ice-cold PBS containing 0.5% formalin and data acquired immediately using the Guava EasyCyte flow cytometer (Guava Technologies, Hayward, CA, USA) and analyzed using the FlowJo software package (Tree Star, Ashland, OR, USA). Cells were identified based on their fluorescent labeling and the YT cells co-labeled with green were considered as conjugates.

### Immunoblotting

For immunoblot analysis, *Cryptococcus* (B3501) was added to YT cells at an E∶T ratio of 1∶100 for 1, 2 and 5 minutes. Cells were lysed in Nonidet P-40 lysis buffer reconstituted with 1× protease inhibitor cocktail and 1× phosphatase inhibitor cocktail (Roche, Quebec, Canada). Lysates were clarified by centrifugation at 18000 g for 5 minutes. After dilution in NuPAGE LDS sample buffer and NuPAGE sample reducing agent, samples were boiled and resolved by PAGE on NuPAGE Bis-Tris gels (Invitrogen Life Technologies), and then transferred to nitrocellulose membranes (Bio-Rad). Immunoblots for phospho-specific Erk1/2 and total Erk1/2 were visualized using the Odyssey infrared imaging system (LI-COR Biosciences) using secondary antibodies according to the manufacturer's instructions. Densitometry was performed using LI-COR Odyssey instrument software.

### Perforin degranulation

NK cells were cultured with or without B3501 (E∶T ratio of 1∶10) in complete medium at pH 7.4 or 6.6 for 48 hours. The cells were harvested, made permeable using BD Cytofix/Cytoperm and washed in BD Perm/Wash buffer according to the manufacturer's protocol. Cells were incubated in 50 µl of staining buffer at 4°C for 20 minutes in the presence of either anti-perforin or isotype-matched control antibodies (both from BD Biosciences). YT cells were selectively identified by labeling with anti-CD11a-PeCy5 antibody (BD Biosciences). Cells were washed in staining buffer (1×PBS with 1% FBS) and fixed with 3.7% formalin in 1×PBS. Flow cytometry was performed using the Guava EasyCyte flow cytometer (Guava) and the data were analyzed using the FlowJo software package (Tree Star, Ashland, OR, USA).

### LAMP-1 surface expression

YT cells (either alone or in the presence of B3501) were cultured in the presence of FITC-conjugated anti-LAMP-1 antibody (BD Biosciences). Golgi-Stop (1 µl) containing monensin (BD Biosciences) was added one hour after the addition of anti-LAMP-1 antibody and incubated for an additional 18 h at 37°C and 5% CO2 in a round bottom 96 well plate. Monensin serves to prevent acidification of the endolysosomal compartment to preserve fluorescence of FITC-conjugated anti-LAMP-1 antibody [61]. At the end of incubation, cells were washed in labeling buffer (1×PBS containing 1% FBS) and fixed with 3.7% formalin in 1×PBS. Flow cytometry was performed using the Guava EasyCyte flow cytometer (Guava) and the data were analyzed using the FlowJo software package (Tree Star, Ashland, OR, USA). YT cells were selectively identified by labeling with anti-CD11a-PeCy5 antibody. Cell surface expression of LAMP-1 on B3501 stimulated YT cell was compared to that of unstimulated YT cells.

### Histopathology

Case 1: Tissue was collected from a cerebral cryptococcoma in a patient who died from cryptococcal (*C.gattii*) meningitis. Case 2: Tissue was collected from a lung nodule in an asymptomatic apparently immunocompetent patient who was exposed to *Cryptococcus* on Vancouver Island, Canada. Tissues were fixed in formalin and processed to paraffin. Sections of these tissues were stained with hematoxylin and eosin, mucicarmine, and PAS. Immunohistochemistry was performed using streptavidin biotin-peroxidase method, according to the manufacturer (ABC Standard Kit, Vector Laboratories Inc., Burlingame, CA). Briefly, after deparaffinization and rehydration, tissue sections were blocked with 3% H_2_O_2_, then with Avidin blocking solution with normal serum for 20 minutes, Biotin blocking solution for 20 minutes (Vector Blocking Kit), followed by overnight incubation at 4°C with anti-CD56, anti-CD57, anti-granzyme B and anti-perforin. A secondary biotinylated anti-Ig was applied followed by horseradish peroxidase conjugated streptavidin. Sections were stained with 3,3-diaminobenzidine (Sigma Fast sets, Sigma Aldrich Chemical Co., St. Louis, MO.) The sections were counterstained with hematoxylin. Nonspecific antibody was used to replace the primary or secondary antibody in negative controls, with tonsillar tissue used as positive control.

### Statistical analysis

The stated numbers of experiments were done on different days using different donors. Values are expressed as mean ± standard error of the mean (SEM) for quadruplicate/triplicate samples. Statistical analysis was performed by ANOVA with Dunnett's post-test, using the Graph Pad Prism version 5 software package (Graph-Pad Software, San Diego, CA, USA). The results of the limiting dilution-based microwell assays were analyzed by the Fisher's exact probability test, which was also performed using the Graph Pad Prism version 5 software package (Graph-Pad Software, San Diego, CA, USA). P value≤0.05 was considered statistically significant.
